# Gualou Xiebai decoction inhibits cardiac dysfunction and inflammation in cardiac fibrosis rats

**DOI:** 10.1186/s12906-016-1012-5

**Published:** 2016-02-04

**Authors:** Yong-fang Ding, Yun-ru Peng, Hong Shen, Luan Shu, Ying-jie Wei

**Affiliations:** 1 Affiliated Hospital of Integrated Traditional Chinese and Western Medicine, Nanjing University of Chinese Medicine, Nanjing, 210028 China; 2 Jiangsu Province Academy of Traditional Chinese Medicine, Nanjing, 210028 China

**Keywords:** GualouXiebai Decoction (GXD), Myocardial fibrosis, Inflammation, NF-κB

## Abstract

**Background:**

Gualou Xiebai Decoction (GXD) is a well-known traditional Chinese recipe. It has been used to treat cardiovascular disorders for nearly two thousand years. But there is a lack of reports on cardiac fibrosis and underlying mechanism.

**Methods:**

Myocardial infarction was performed by ligation of left anterior descending coronary artery (LAD) in male Wistar rats. Rats with myocardial infarction were treated with GXD (1.14 g/kg, 4.53 g/kg) daily for 4 weeks. Cardiac function was evaluated by echocardiography. Hemodynamic parameters and infarct size were measured in each group. Myocardial enzymes were examined by biochemical tests. Inflammatory cytokines were assessed by ELISA, and interrelated proteins were detected by western blot.

**Results:**

Cardiac function was significantly improved in GXD-treatment rats after myocardial infarction (MI), which was accompanied with decreased infarct size. Administration of GXD to myocardial fibrosis rats significantly ameliorated the activities of AST, LDH and CK-MB in serum. The increase in inflammatory factors (TNF-α, IL-1β) were markedly reduced upon GXD treatment. Furthermore, the inflammatory mediators (NF-κB p65, TNF-α, MCP-1) were down-regulated by GXD in the myocardial fibrosis rats.

**Conclusions:**

Treatment with GXD improved cardiac function induced by myocardial fibrosis by inhibiting expression of inflammatory mediators associated with NF-κB.

## Background

Gualou Xiebai Decoction (GXD), also known as Gualou Xiebai Baijiu Decoction, is a preparation consisting of *Trichosanthes kirilowii* Maxim and *Allium macrostemon* Bge in a weight ratio of 2:1. This formula is firstly described by Zhang Zhong-jing (in the Chinese Donghan Dynasty, 3 century) in the treatise “Jin Kui Yao Lue”. For centuries, GXD has been widely used to treat many cardiovascular disorders, including myocardial infarction, heart failure, and arrhythmias [1, 2].

Myocardial fibrosis, is thought to contribute to sudden cardiac death, ventricular tachyarrhythmias, left ventricular dysfunction, and heart failure.It is characterized by a structural rearrangement of the cardiac chamber wall that involves cardiomyocyte hypertrophy, fibroblast proliferation, and increased deposition of extracellular matrix (ECM) proteins [3]. The proliferation of interstitial fibroblasts and increased deposition of ECM components results in myocardial stiffness and diastolic dysfunction, which ultimately leads to heart failure [4]. Fibrosis typically results from activation of inflammation and reparative pathways in response to a persistent injurious stimulus. Most types of cardiac injury trigger a local inflammatory reaction of proteases. Therefore, it is important to examine the effect of GXD on inflammatory response contributing to myocardial fibrosis.

Our previous studies had shown that ethanol extraction from GXD could ameliorate myocardial fibrosis by reduced left ventricle weight/body weight ratio, prevented the expression of Collagen I and Collagen III, the mechanism maybe involve in inhibiting the TGF-β1 signaling pathway [5]. However, it remains unclear how GXD has an effect on cardiac function and heart remodeling after myocardial infarction. It is suggested that activation of inflammatory mediators related to NF-kappa-B (NF-κB) could be major components of myocardial fibrosis [6]. Therefore, the aim of this study was to provide additional evidence of its effect on alterations of cardiac function and heart remodeling in myocardial fibrosis rats, as well as the effects of GXD on cardiac inflammation relevant to myocardial fibrosis.

## Methods

### Plant material

Gualou and Xiebai were identified and authenticated as whole fruits of *Trichosanthes kirilowii* Maxim and dried bulbs of *Allium macrostemon* Bunge by Professor Qian Shi-hui (Jiangsu Province Academy of Traditional Chinese Medicine, Nanjing, China). A voucher specimen (2012-01-08) was deposited in the herbarium of this academy.

### Extract preparation

Gualou (*Trichosanthes kirilowii* Maxim) and Xiebai (*Allium macrostemon* Bge) were mixed in a ratio of 2:1, reaching a total weight of 4000 g. The mixture was decocted three times under refluxing with 50 % ethanol, each for 2 h. The solution obtained was concentrated to dryness on a rotary vacuum evaporator, affording 1509.6 g extract (yield: 37.74 %). The main compounds of the extract were identified by UPLC-QTOF/MS/MS as reported previously [5].

### Ethics statement

Animal experiments were performed in accordance with the *Guide for the care and Use of Laboratory Animals* published by the US National Institutes of Health (publication no. 85–23, revised 1996), and the study was approved by our Institute Animal Experimental Ethical committee. And the performed of experiment was fit the international, national and institutional animal experiment rules. All the animals were sacrificed by anesthesia at the end of the experiment.

### Experimental animals

Male Wistar rats (*n* = 54) weighing 250–300 g were purchased from Sino-British Sippr/BK Lab Animal Ltd. (Shanghai, China). The rats were housed under a 12 h: 12 h light/ dark cycle, with free access to standard rat-chow diet and tap water. Animals were allowed a 1-week acclimatization period prior to the experimental protocol.

### Myocardial infarction model

Rats were intraperitoneally anesthetized with 10 % chloral hydrate (300 mg/kg), intubated with polyethylene tube and ventilated with an automatic breathing apparatus (Shanghai Alcott Biotech CO., LTD., China) at a respiratory rate of 70 breaths/min and a tidal volume of 2.0 ml. The heart was rapidly exteriorized. A 6–0 silk suture was placed through the myocardium into the anterolateral left ventricular wall around the left anterior descending coronary artery (LAD) approximately 2–3 mm distal from its origin. The vessel was permanently ligated. The ligation was confirmed successfully when the anterior wall of the left ventricle turned pale. After surgery, the chest was closed and the animals were allowed to recover. A similar surgery procedure was performed without LAD ligation in 8 rats (Sham group) [7]. Ten rats died during the 24-h postoperative period because of acute pumps failure or lethal arrhythmias. 36 rats with myocardial infarction (MI) survived.

The 36 rats with MI were randomly divided into three groups of 12 rats, each divided by treatment with high- or low-dose GXD, or distilled water: GXD rats received low doses of GXD (1.14 g/kg, oral gavage), or high doses (4.53 g/kg, oral gavage), and MI rats received distilled water as did Sham rats. These doses were selected on the basis of human clinical dosage and our earlier studied of the cardio protective effect of GXD; the appropriate dose for the GXD extract was calculated according to the yield [8, 9]. Treatment with GXD was continued further for 4 weeks once daily at the same time.

### Echocardiographic measure of cardiac function

To evaluate LV (left ventricular) function, transthoracic echocardiography was performed with a Vevo 2100 instrument (VisualSonics) at the end of the study [10]. Under general anesthetized with chloral hydrate, animals were imaged using a 40-MHZ linear array transducer. M-mode and two-dimensional parasternal short-axis scans at the level of the papillary muscles were used to assess changes in LV end-diastolic inner diameter (LVID;d), LV end-systolic inner diameter (LVID;s), LV posterior wall thickness in end-diastole (LVWP;d) and in end-systole (LVPW;s) and interventricular septal diastolic wall thickness (IVS; d) and systolic wall thickness (IVS;s). To assess LV systolic function, fractional shortening (FS) and ejection fraction (EF) were calculated as follows: FS = [(LVID;d - LVID;s)/LVID;d] × 100 %, EF = [(LVID;d^3^-LVID;s^3^)/LVID;d] × 100 %.LV end- diastolic volume (LVEDV) and end-systolic volume (LVESV) were acquired by 7/(2.4 + LVID; d) × LVID; d^3^ and 7/(2.4 + LVID; s) × LVID; s^3^, respectively.

### Hemodynamic measurement

To determine the heart hemodynamic function, parameters were measured in closed-chest anesthetized rats [11]. Animals were anesthetized with chloral hydrate and trachea was intubated to facilitate breathing. Body temperature was maintained at 37 to 38 °C. A midline incision was made to expose the right common carotid artery. A catheter tip pressure transducer (Mikrotip 1.4 F, Millar Instrument, Houston, TX, USA) was then inserted into the right carotid artery and advanced to the LV under guidance of the pressure signal and fixed in position. The heart rate, systolic and diastolic pressure, LV pressure, and the rate of pressure development (maximal positive, +dP/dt; maximal negative, −dP/dt) were recorded at a rate of 500 HZ through a Biopac MP100 data acquisition unit (Biopac systems Inc., Goleta, CA, USA). A period of approximately 20 min was allowed for stabilization of cardiovascular parameters before the hemodynamic data were recorded. An 8 s recording was analyzed with AcqKnowledge software for each animal.

### Myocardial enzymes and inflammatory cytokines measurement in serum

After hemodynamic measurements, the blood samples were collected from the abdominal artery and the serum was separated. Creatine kinase-MB (CK-MB), aspartate aminotransferase (AST) and lactate dehydrogenase (LDH) were determined by auto biochemical analysis system (Roche Modular DP, Germany). Interleukin-1beta (IL-1β) and tumor necrosis factor-alpha (TNF-α) in serum were determined by enzyme-linked immunosorbent assay (ELISA) kits (R&D ELISA Kit, USA) following the manufacturer’s instructions.

### Infarct size evaluation

To evaluate the infarct size, the hearts were removed and dissected. Cardiac tissue samples were fixed in a 10 % formaldehyde solution for 48 h and stained with hematoxylin and eosin according to the previously described method [12]. The infarction size of left ventricle was determined in sections 5 to 6 mm from the apex, since the values in this region correspond to the mean of the values obtained from sections of the entire heart. The lengths of the infracted and viable myocardial for both the endocardial and epicardial circumferences were determined by planimetry [13]. Infarction size was expressed as a percentage of the sum of endocardial and epicardial infracted ventricular lengths to the sum of total (infracted and viable myocardial) endocardial and epicardial ventricular circumferences. Slides were observed with the Zessi Axioskop 2 microscope and digitally photographed, and analyzed using Image-Pro Plus 6.0 (Media Cybernetics Inc., Baltimore, MD, USA).

### Western blot analysis

Nuclear protein was extracted by Nuclear Extraction Kit (Millipore) and total cellular protein was extracted from frozen tissue by RIPA buffer containing 1 mM PMSF. All protein extractions were adjusted to equal contraction using BCA assay. Equal amounts (40 μg/lane) of each sample were separated on polyacrylamide gel electrophoresis and transferred by electrophoresis onto nitrocellulose membrane (Millipore). Following washing and blocking, the membrane was incubated overnight at 4 °C with primary antibody against NF-κB p65 (Santa Cruze, 1:1000), TNF-α(Santa Cruze, 1:1000), MCP-1(Santa Cruze, 1:1000). After washing, the membrane was incubated with secondary antibody at 37 °C for 1 h. Bands were visualized by enhanced chemiluminescence kit (Pierce, Rockford, IL, USA). Target proteins levels were normalized byGAPDH.

### Statistical analysis

Data are expressed as mean ± SD. All statistical analyses were performed using SPSS software (Version 11.5, SPSS Inc., Chicago, IL, USA). Statistical significance involving multiple groups was performed using a one-way analysis of variance (ANOVA). Differences were considered to be significant at *P <* 0.05.

## Results

### GXD improved cardiac function caused by cardiac fibrosis

**Fig. 1:**
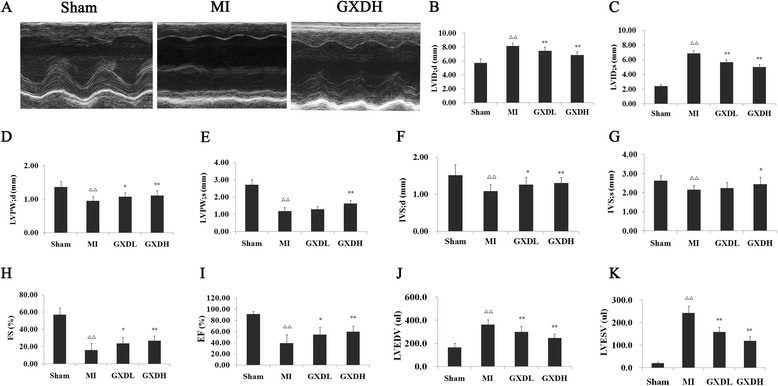
Effect of GXD on echocardiographical parameters in MI rats. **a** Representative echocardiography pictures of M mode images. The quantitative data of (**b**) LV end-diastolic inner diameter (LVID; d); (**c**) LV end-systolic inner diameter (LVID; s); (**d**) LV posterior wall thickness in end-diastole (LVWP; d); (**e**) LV posterior wall thickness in end-systole (LVPW; s); (**f**) interventricular septal diastolic wall thickness (IVS; d); (**g**) interventricular septal systolic wall thickness (IVS; s); (**h**) fractional shortening (FS); (**i**)ejection fraction (EF); (**j**) LV end- diastolic volume (LVEDV); (**k**)end-systolic volume (LVESV). Results are expressed as mean ± S.D. (*n =* 8 for Sham group and *n =* 12 for other groups). ^△△^ *P <* 0.01 *vs* sham group; *P < 0.05, **P < 0.01 *vs* MI group

Cardiac function is known to deteriorate at later stages after MI and is, in fact, becoming an increasingly more frequent cause of cardiac failure in post-MI survivors. To determine whether GXD transfer improved cardiac performance, we performed echocardiography four weeks after MI (Fig. 1). LVID;d and LVID;s were significantly increased in MI rats (*P* < 0.01, Fig. 1b, c) relative to sham rats, and a marker decrease in low- and high-dose groups (*P* < 0.01, *vs* MI group). IVS and LVPW at both systole and diastole exhibited lower in MI group than Sham group (*P* < 0.01, Fig. 1d, e, f, g), and 1.14 g/kg and 4.53 g/kg groups showed higher IVS and LVPW than that of MI group (*P* < 0.05 ~ 0.01), which indicated that GXD could inhibit interventricular septal hypertrophy. FS and EF, the index of left ventricular systolic function, were significantly decreased in MI group, these changes were attenuated when MI rats were treated with GXD (*P* < 0.05 ~ 0.01, *vs* MI group, Fig. 1h, i). Furthermore, LVEDV and LVESV were greater in MI group compared with Sham group respectively (*P* < 0.01, Fig. 1j, k), and they were smaller in low- and high-dose groups (*P* < 0.01, *vs* MI group). These data suggest that GXD could attenuate myocardial dysfunction induced by myocardial fibrosis. To further assess cardiac function, hemodynamic parameters were analyzed four weeks after GXD-treated. After MI, LVEDP was increased, while HR, SBP, DBP, LVSP were decreased in MI rats when compared with sham-operated rats (*P <* 0.01, Table 1). Treatment with GXD almost restored all of them to the near normal levels (*P <* 0.05 ~ 0.01, vs MI group). There were significant decrease in ± dP/dt between the Sham group and MI group (*P <* 0.01, Table 1). However, the decrease in ± dP/dt in MI rats were significantly increased by GXD treatment (*P <* 0.05 ~ 0.01, *vs* MI group). The significant changes of SBP, DBP, LVSP, LVEDP and ± dP/dt indicated that GXD could improve cardiac function of post-infarction.

**Table 1:** Effect of GXD on hemodynamic parameters and cardiac function induced by coronary artery ligation

	sham	MI	GXDL	GXDH
HR(beats/min)	359.6 ± 42.3	259.8 ± 29.5^△△^	306.0 ± 55.4^*^	333.7 ± 57.2^**^
SBP(mmHg)	107.1 ± 10.1	82.9 ± 7.7^△△^	88.4 ± 7.3	99.1 ± 10.2^**^
DBP(mmHg)	69.93 ± 10.47	56.48 ± 8.07^△△^	62.59 ± 5.26^*^	67.26 ± 11.65^*^
+LV dp/dt_max_(mmHg/s)	5287.03 ± 1247.53	3312.07 ± 859.48^△△^	4062.42 ± 623.19^*^	4752.61 ± 930.10^**^
-LV dp/dt_min_(mmHg/s)	4099.12 ± 759.61	3005.07 ± 517.25^△△^	3357.75 ± 774.67	3822.46 ± 905.39^*^
LVSP(mmHg)	120.99 ± 10.15	88.94 ± 6.86^△△^	95.70 ± 7.57^*^	105.24 ± 9.85^**^
LVEDP(mmHg)	−4.57 ± 1.71	5.72 ± 1.44^△△^	2.69 ± 0.88^**^	−0.98 ± 1.70^**^

Values are expressed as the mean ± S.D. (*n =* 8 for Sham group and *n =* 12 for other groups). *HR* heart rate, *SBP* systolic blood pressure, *DBP* diastolic pressure, *±LV dp/dt*_*max*_ the maximal rates of increase and decrease of left ventricle pressure development, *LVSP* left ventricular systolic pressure, *LVEDP* left ventrilcel end dilated pressure. ^△△^ *P <* 0.01 *vs* sham group; *P < 0.05, **P < 0.01 *vs* MI group

### GXD inhibited myocardial injury and inflammation

**Fig. 2:**
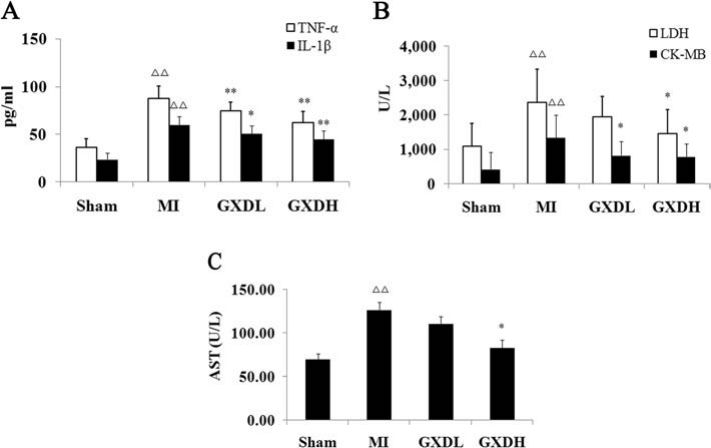
Effect of GXD on myocardial injury and inflammation factors. The quantitative data of (**a**) aspartate aminotransferase (AST); (**b**) lactate dehydrogenase (LDH) and Creatine kinase-MB (CK-MB); (**c**) tumor necrosis factor-alpha (TNF-α) and Interleukin-1beta (IL-1β). Results are expressed as mean ± S.D. (*n* = 8 for Sham group and *n =* 12 for other groups). ^△△^ *P <* 0.01 *vs* sham group; *P < 0.05, **P < 0.01 *vs* MI group

Myocardial enzymes CK-MB, AST, LDH are biochemical indicators of myocardial injury. As compared with Sham group, the levels of all three enzymes were significantly elevated in the MI group (*P <* 0.01, *vs* Sham group, Fig. 2a, b). GXD protected MI rats against cardiac injury, which was evidenced by decreased myocardial enzymes in GXD-treated group (*P <* 0.05 ~ 0.01, *vs* MI group).

Moreover, the inflammatory factors IL-1βand TNF-αwere also increased in MI group compared to Sham group (*P <* 0.01, Fig. 2c). By contrast, lower levels of IL-1βand TNF-α were found in GXD-treated group compared to MI group (*P <* 0.05 ~ 0.01).

### GXD reduced infarct size after MI

**Fig. 3:**
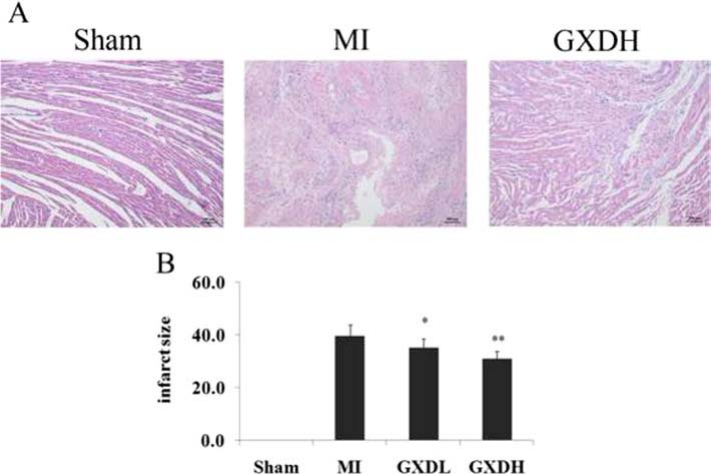
Effect of GXD on infarct size. **a** Representative microphotographs (×100) stained with hematoxylin and eosin (H/E). **b** Histogram of the effect of GXD on infarct size. Results are expressed as mean ± S.D. (*n =* 8 for Sham group and *n =* 12 for other groups). *P < 0.05, **P < 0.01 *vs* MI group

To substantiate the effect of GXD in LV remodeling after MI, we analyzed infarct size. Quantitative evaluation of infarct size revealed that there was significant decrease in infarct size in MI group compared with the sham group (*P <* 0.01; Fig. 3). This decrease in infarct size was prevented by treatment with GXD (*P <*0.05 ~ 0.01, *vs* MI group).

### GXD inhibited inflammatory mediators in the MI heart to prevent myocardial fibrosis

**Fig. 4:**
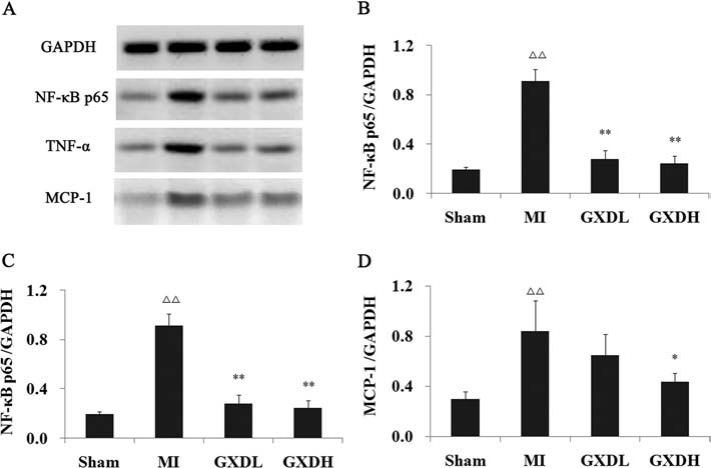
Effect of GXD on inflammatory mediators expression. **a** Representative Western blotting analysis of myocardial fibrosis tissue. The ratios of various proteins to GAPDH are presented in panels **b**, **c**, **d**. Protein levels were calculated by densitometry. Results are expressed as mean ± S.D. (*n =* 3). ^△△^ *P <* 0.01 *vs* sham group; **P < 0.01 *vs* MI group

The NF-κB family plays an important role in inflammatory responses by promoting the expression of proinflammatory factors, we investigated the NF-κB p65 by which GXD attenuates myocardial fibrosis. As shown in Fig. 4, the protein levels of p65 protein in the nucleus was significantly increased in the MI heart (*P* < 0.01 *vs* Sham group) but reduced in the GXD-treated MI heart (*P* < 0.01, *vs* MI group). In line with the alteration of the NF-κB signaling, NF-κB-targeted cytokines, such as TNF-α and MCP-1 expression, were significantly elevated in the MI heart. However, their levels were much lower in the heart of GXD-treated MI hearts.

## Discussion

Generally, cardiac function requires the orchestration of multiple mechanical and biological factors within the myocardial microenvironment. The cellular matrix provides a structural foundation for the myocytes, fibroblasts, and endothelial cells to build an architectural network and provides an environment for cell signaling, cell-cell interactions, etc. [14, 15]. Excessive myocardial fibrosis is an important pathophysiological process that contributes to diastolic and eventually systolic dysfunction by increasing myocardial stiffness and reducing pumping capacity. The amount of collagen present in the myocardium has also been reported to represent the most significant factor related to echocardiographic demonstration of diastolic dysfunction in hypertensive heart disease [16, 17]. In this report, we have demonstrated that GXD could attenuate LV systolic and diastolic functional echocardiographic and hemodynamic parameters induced by myocardial fibrosis.

Fibrosis typically results from activation of inflammatory and reparative pathways in response to a persistent injurious stimulus [18]. Most types of cardiac injury trigger a local inflammatory reaction inducing upregulation of cytokines and fibrogenic growth factors and promoting activation of proteases [19]. Myocardial infarction results in cardiomyocyte necrosis and induces an intense inflammatory response. The cardiac repair process can be divided into three overlapping phases: the inflammatory phase, the proliferative phase and the maturation phase. During the inflammatory phase, cardiomyocyte death and hypoxia result in free radical generation, initiation of the complement cascade, and activation of NF-κB and monocyte chemoattractant protein-1 (MCP-1)/CCL-2 signaling pathway [19, 20]. Both macrophage and T cells may release cytokines that can act on cardiac resident cells during the inflammation processes of infarct healing. Among the release cytokines are interleukins (ILs), tumor necrosis factors (TNFs), interferons (IFNs), platelet-derived growth factor (PDGFs), fibroblast growth factors (FGFs), transforming growth factor (TGF)-βs, etc.[21].

NF-κB, which regulates inflammatory and immune processes, is a ubiquitous transcription factor. Members of NF-κB family (p50, p52, p65, c-Rel and Rel B) form homo or heterodimers (most commonly p50/p65, p50/p50, or p65/p65) that are bound to inhibitory IκB proteins in the cytosol [22, 23]. Once activated, NF-κB translocates into the nucleus from the cytosol, then binds to the consensus sequence on promoter or enhancer regions of related genes and regulates gene transcription [24]. NF-κB plays an important role in myocardial fibrosis. NF-κB contributes to myocardial fibrosis pathogenesis because it regulates genes/proteins important for disease progression, including cytokines (e.g., TNF-α), interleukins (e.g.,IL-1β). TGF-β/Smads signaling are markedly induced in the infracted myocardium and through their potent effects are capable of playing a central role in infarct healing, cardiac repair and left ventricular remodeling. TGF-β1 overexpressing mice exhibited significant cardiac hypertrophy accompanied by interstitial fibrosis [25]. It is now well accepted that inactivation of Smad2/3 by overexpression Smad7 inhibits NF-κB –dependent inflammatory response by inducing inhibitor of NF-κBα expression. So, the interaction between the TGF-β/Smads and NF-κB signaling pathways may be important in cardiac inflammation [26, 27]. Previously, we have showed that GXD ameliorated heart injury and protected rats from myocardial fibrosis, which may be related to inhibiting the TGF-β1 signaling pathway by down-regulating expressions of Smad2/3 whereas improving Smad7 expression. And in this paper, we found that GXD could down-regulate NF-κB and NF-κB-targeted cytokines, such as TNF-α and MCP-1 expression. The further studies are necessary to elucidate the effect of GXD on cross-talk of TGF-β and NF-κB signaling.

## Conclusions

We demonstrated that myocardial fibrosis induced by ligating the left anterior descending coronary artery was clearly prevented by treatment with GXD. Cardiac function was significantly improved in GXD-treatment rats after MI (myocardial infarction). From molecular analyses, we concluded that the effect of GXD were dependent on inhibition of the inflammatory response through the NF-κB pathway. It could dose-dependently improve the cardiac function after MI by downregulation of NF-κB expression, and suppressing NF-κB-targeted cytokines, such as TNF-α and MCP-1 production. However, the effect of GXD on restraining myocardial fibrosis still needs further investigation.
